# Prey localization in spider orb webs using modal vibration analysis

**DOI:** 10.1038/s41598-022-22898-3

**Published:** 2022-11-09

**Authors:** Martin Lott, Vinicius F. Dal Poggetto, Gabriele Greco, Nicola M. Pugno, Federico Bosia

**Affiliations:** 1 Department of Applied Science and Technology, Politecnico di Torino, Torino, Italy; 2 Laboratory for Bioinspired, Bionic, Nano, Meta Materials and Mechanics, University of Trento, Trento, Italy; 3 School of Engineering and Materials Science, Queen Mary University of London, Mile End Road, London, E1 4NS United Kingdom

**Keywords:** Bioinspired materials, Mechanical properties

## Abstract

Spider webs are finely tuned multifunctional structures, widely studied for their prey capture functionalities such as impact strength and stickiness. However, they are also sophisticated sensing tools that enable the spider to precisely determine the location of impact and capture the prey before it escapes. In this paper, we suggest a new mechanism for this detection process, based on potential modal analysis capabilities of the spider, using its legs as distinct distributed point sensors. To do this, we consider a numerical model of the web structure, including asymmetry in the design, prestress, and geometrical nonlinearity effects. We show how vibration signals deriving from impacts can be decomposed into web eigenmode components, through which the spider can efficiently trace the source location. Based on this numerical analysis, we discuss the role of the web structure, asymmetry, and prestress in the imaging mechanism, confirming the role of the latter in tuning the web response to achieve an efficient prey detection instrument. The results can be relevant for efficient distributed impact sensing applications.

Many natural structures have fascinated and inspired researchers over the years for their optimized characteristics. Spider webs are a particularly prominent example, due to their various functions, their versatility and efficiency. Spider webs occur in different shapes and geometries, and allow arachnids to perform a vast variety of tasks such as capturing prey, lifting heavy objects and throwing themselves at accelerations around 80g^1,2^. Of all the types of webs, however, two dimensional orb webs are probably the most renowned. These particular structures are typically composed of radial and frame threads produced with major ampullate silk^3^, which are secured to surfaces through the cement-like piriform silk^4^. The radial threads are intercepted with the sticky spirals threads of the web that are produced with aggregate and flagelliform silk and efficiently adhere to the prey^5–8^.

From a mechanical point of view, spider webs display multiple excellent properties in terms of strength and toughness of the single silk threads, adhesion of the anchorages with the surfaces, and impact resistance^9^. These web characteristics also translate into an outstanding mechanical efficiency in localizing and catching prey^10,11^, a process that benefits from the mechanical properties of the silk and the vibration properties of the web^12,13^. Recent studies based on transient response analysis have indicated that prestressing is used to tune the sonic properties of webs^13,14^, which has a great influence on flexural wave propagation that may be used by spiders as a means to localize prey^15,16^. Laser vibrometry measurements have shown that radial threads are able to efficiently transmit the entire frequency range between 1 and 10 kHz^17,18^, frequencies that are detected by the cuticular vibration receptors of the spiders^19^. These threads can undergo transverse (perpendicular to the thread and the plane of the web), lateral (perpendicular to the thread and in the plane of the web), and longitudinal (along the thread axis) motions^17^. The propagation of transverse and longitudinal waves are governed by distinct mechanisms^20–22^, giving rise to different propagation speeds and damping mechanisms^12,18,23^. The overall transmitted frequency by a web may vary from around 100 Hz, which is typical of insect impacts, to peaks ranging from 5 Hz to 50 Hz due to the leg movements of trapped insects, and between 100 Hz and 300 Hz due to fluttering bees and flies. The frequency contents of such signals differ considerably from those that may arise due to wind loading, typically below 10 Hz. The unique wave transmission characteristics of spider orb webs must therefore be able to act as mechanical filters for a wide variety of inputs.

In this work, we discuss a possible vibration detection mechanism used by a spider to sense prey impacts using modal decomposition of the web motion. Vibrations are one of many physical quantities used by spiders for sensing^15^. Without wishing to address the details of the biology of spider sensing, here, we aim to demonstrate through mathematical and physical considerations the feasibility of using vibrations in a spider orb web for effective prey impact localization, and its compatibility with existing experimental data^6^. We use the modal expansion of Green’s function to construct a semi-analytical source signal created by an impact. Then, the visibility of the spider to this signal is addressed with Green’s function composition methods, allowing the web motion to be analyzed at the location of the spider legs. This imaging method has recently been validated with experiments on thin elastic plates^24^. In the present study, the designed numerical web uses prestressed truss elements and nonlinear geometry effects to model realistic transverse elastic properties for such structures. We show that this approach can clearly highlight the influence of prestress, which drives the transverse rigidity; we also assess the role of imperfections within the web geometry, which help to split the degenerate modes of the structure; finally, we discuss the role of the spider position in terms of the overall imaging capabilities of the spider/web system. This work complements an extensive body of evidence that documents the optimization of structures for vibration control found in Nature^25^.

## Materials and methods

### Spider web geometry

**Figure 1:**
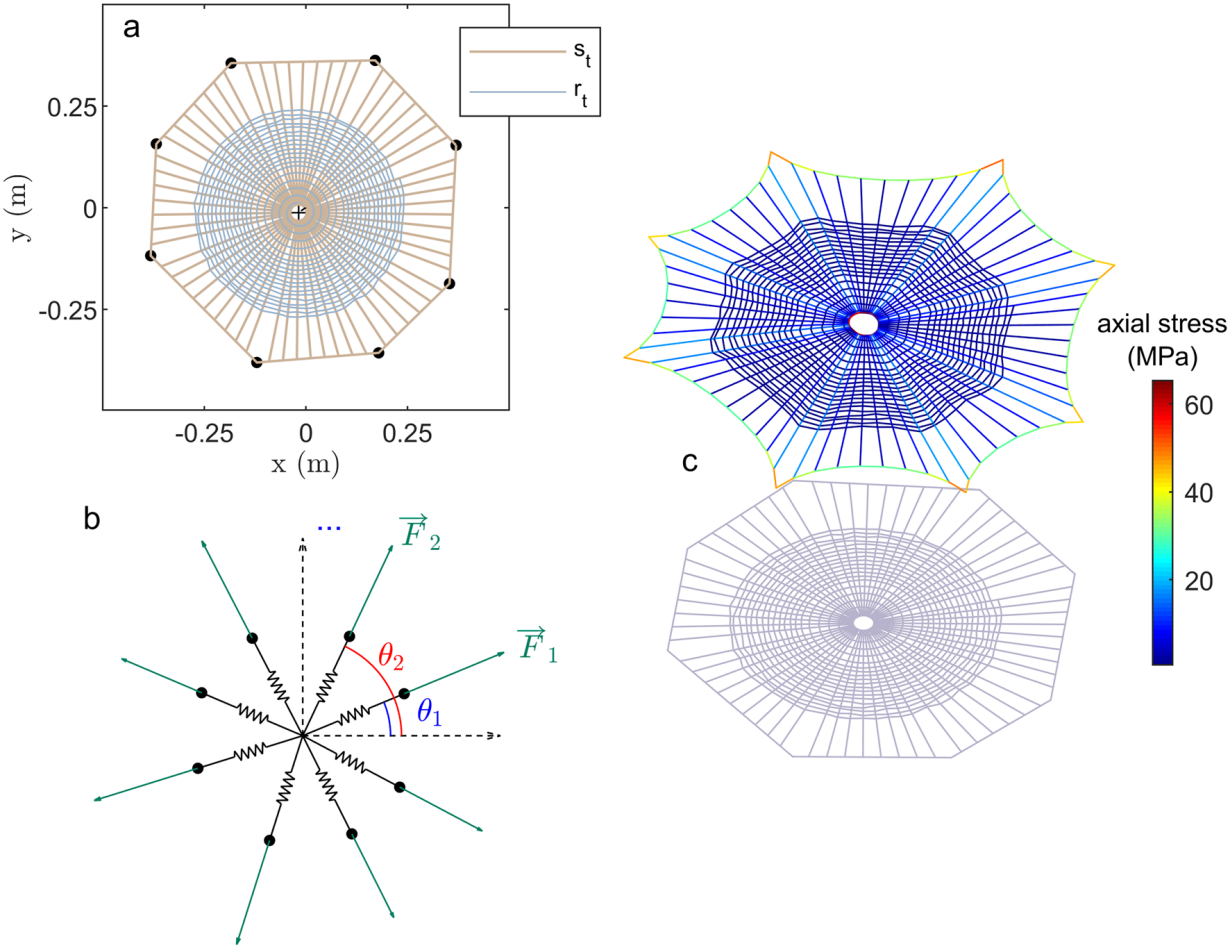
(Color online) Design of the orb web. Light blue lines are the *spiral threads* $r_t$. The light brown line is the *radial thread* $s_t$. (**a**) Initial, unstressed geometry. (**b**) Radial force distribution in the web. The equilibrium point should satisfy $\Sigma _i {\mathbf {F}}_i = {\mathbf {0}}$, with *i*=1..8. (**c**) Final equilibrium configuration considering geometrical nonlinearity and stress values in color scale. Pictures and maps are made with $\hbox {COMSOL}^{{\mathrm{TM}}}$ 6.0 and $\hbox {MATLAB}^{{\mathrm{TM}}}$ R2022b softwares https://www.comsol.com/livelink-for-matlab.

The orb web model is built using thin threads, combining various structural elements in a deliberately asymmetrical (i.e. more realistic) assembly. The first element is a structural frame that surrounds the web, built in an irregular octagonal shape, included in a 40 cm diameter circle (the 8 corner points of the octagon are at equal distances from the center). The second element of the assembly is an ensemble of radial threads built from an off-center small circle inside the previously designed octagonal shape, connected to the surrounding structure (light brown in Fig. 1a) with a constant angular distribution of the threads. The third element of the assembly are the spiral threads (light blue in Fig. 1a), built as a spiral of maximum radius of 30 cm, starting at the small off-center circle, with an angle-varying radius, allowing a smaller step in the outer part of the spiral than in the center, with an additional randomness value, conferring a more realistic appearance to the obtained orb web. The overall resulting geometry is depicted in Fig. 1a. This is the original, unstrained geometry, i.e., before any applied prestress.

### Simulation box

The web structure is mechanically modeled with the Finite Element software COMSOL Multiphysics using prestressed truss elements, with the inclusion of geometrical nonlinearity effects. Prestress values induce a “stress stiffening” effect, where the transverse elasticity of a thread is directly proportional to the tensile force acting on it. Geometrical nonlinearity allows the inclusion of large strain values and geometrical deformations. Similar to the case of a string, if the axial stress is zero or negative, the transverse stiffness of a truss is zero^26^. The final stretched web acts, therefore, as a transverse resonator. The overall modal response of the web is investigated using eigenvalue analysis. The numerical modeling is thus based on the hypothesis that the original nonlinear problem can be treated as a linear eigenvalue problem: despite the large displacement amplitude induced by the prestress and geometrical nonlinearity, the modal vibrations occur around this new deformed state with small vibration amplitudes (i.e., dynamic displacements are small compared to the prestress-induced static displacements). This means that the dynamic amplitude does not affect the rigidity of the structure, which is essentially due to static forces. In order to include a representative mechanical response of the numerically designed orb-web, we associate for the surrounding frame and axial threads elements (light-brown color in Fig. 1a) and to the spiral threads (light-blue color in Fig. 1a) the mechanical and structural parameters indicated in Table 1.

**Table 1:** Input mechanical parameters for the orb web simulations in its initial state. Two types of threads (Radial/Surrounding and Spiral) are mechanically modeled with different parameters.

Threads	$\varnothing$ ($\mu$m)	E (MPa)	$\nu$	$\rho$ (kg/$\hbox {m}^{-3}$)	$\sigma _0$ (kPa)
Radial/surrounding	5	100	0.1	1350	10
Spiral	3	1	0.1	1350	10

From^27^, the nonlinear stress/strain law for the silk suggests a non-constant value for the Young’s modulus, with an early transition to plasticity. The use of realistic stress values and elastic properties for the silk requires the integration of a complete nonlinear material law in order to obtain the global deformation and stress of the total structure observed in Nature. In the present study, we use the Young’s modulus value in the plastic zone, i.e., around 100 MPa. In this manner, the final stress value supported by the thread is coherent with previously published results^28^, with no need to add more complexity to the simulation box. In addition, the selected value for the Poisson’s ratio does not influence the simulations, as the transverse elastic properties of the web are only determined by the stress values in the truss and thus, by its Young’s modulus.

### Static study

The structure is initially stretched from the eight points that surround the structure (black dots in Fig. 1a) by a set of pre-determined forces, acting from the center of the web to the exterior. As the pulling points are not equally spaced, the set of forces needs to be determined so that their sum is zero, to avoid rigid-body motion. The following expression for the set of forces acting on the web is considered,

1 $$\begin{aligned} \overrightarrow{F}_i = F_0 \times \left( x_i\cos (\theta _i)\overrightarrow{e}_1 + x_i\sin (\theta _i)\overrightarrow{e}_2\right) \end{aligned}$$

where $x_i$ values are the equilibrium parameters that we choose to fix in the ]0.81.2[ interval and $F_0$ is the global force amplitude. Projection on the x and y axes of this cumulative set of forces should be zero, which leads to the following cost function to be minimized:

2 $$\begin{aligned} g({\mathbf {x}}) = \left| \Sigma _i x_i \cos \left( \theta _i\right) \right| + \left| \Sigma _i x_i \sin \left( \theta _i\right) \right| \end{aligned}$$

Figure 1b provides the graphical representation of this set of forces after determining the 8 parameters $x_i$ in a geometrical frame, centered on the radial thread origin (off-center circle). To find the solution, we use a stochastic population-based algorithm (i.e., a genetic algorithm) that searches randomly by mutation and crossover among population members. The solution is not unique, however, once one set of $x_i$ is found, the global force amplitude can be adapted with the $F_0$ parameter.

After applying with the calculated forces on the structure with a 2 mN amplitude for the $F_0$ value, the final geometrical state of the web differs from initial one. In Nature, a spider will construct the radial threads and apply a stress to them before adding the spiral threads. This differs from our procedure where the entire structure is built simultaneously. However, this should not influence the numerical results significantly. To prevent local compression arising during the pulling phase of the web, an initial and homogeneous prestress is globally applied to all of the threads of the structure with a small value (additional to the final stress) of $\sigma _0 =$ 10 kPa. The final geometry and stress state for the structure are depicted in Fig. 1c. The curved profiles of the threads that appear around the web are due to nonlinear geometrical effects. The stress distribution inside the web is influenced by both global prestress and applied forces. A high level of stresses (up to 60 MPa) is reached.

### Modal decomposition

**Figure 2:**
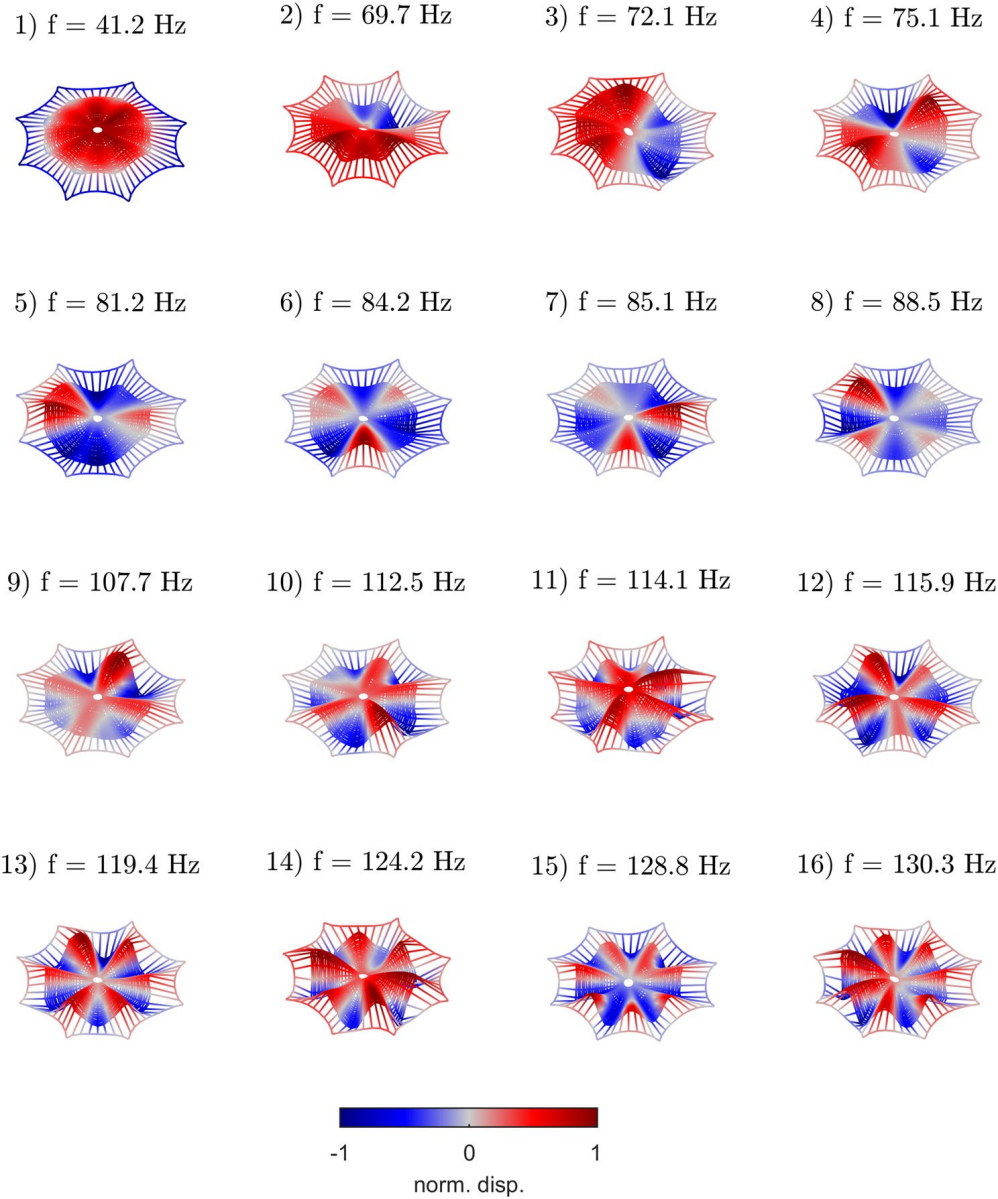
(Color online) Vibration eigenmodes of the spider web, modeled by pre-stressed trusses with geometrical nonlinearity. Pictures and maps are made with $\hbox {COMSOL}^{{\mathrm{TM}}}$ 6.0 and $\hbox {MATLAB}^{{\mathrm{TM}}}$ R2022b softwares.https://www.comsol.com/livelink-for-matlab.

Eigenfrequency analysis is performed on the prestressed structure, after the quasi-static traction. The final outputs for this study step are then the resonant frequencies $f_n$ and the orthonormal modal shapes $\phi _{in}$, where *n* indicates the mode index, and *i* a mesh point index in the discretized and unstrained web domain. Both quantities are dependent of the applied forces from the previous study. Summing over repeated *i* indices, the modal orthogonality implies that $\phi _{ni}^{\scriptscriptstyle \dag }\phi _{in'} = \delta _{nn'}$ where $(\cdot )^{\scriptscriptstyle \dag }$ indicates the complex conjugate transpose and $\delta _{nn'}$ is the Kronecker delta function. Figure 2 shows the first 16 eigenmodes of the simulated structure in a prestressed state. The image displays the motion of the orb web in the deformed geometry frame considering the transverse normalized displacement field. The frequency range of the modes predicted with the simulation box agrees with in-situ experimental work on real orb webs^29,30^.

To highlight the limitations of neglecting the changes in geometry in the eigenvalue analysis, we can consider the analytical expression of the fundamental resonant frequency for a string:

3 $$\begin{aligned} f_0 = \dfrac{1}{2L}\sqrt{\dfrac{T}{\rho _l}} \end{aligned}$$

where *L* is the string length, $\rho _l$ is its mass per unit length, and *T* the tension acting on it. If the pulling force increases, the relative change of frequency $f_0$ can be estimated (neglecting the changes in the bulk density due to deformation) as follows:

4 $$\begin{aligned} \dfrac{\delta f_0}{f_0} = - \dfrac{\delta L }{L} + \dfrac{1}{2} \dfrac{\delta T }{T} \end{aligned}$$

As a consequence, two opposite effects appear when increasing the pulling forces on the web: an increase of stress that leads to an increase stiffness and hence resonant frequency, and an increase of strain, i.e. in the structure length, which tends to decrease the resonant frequency. This last term is not taken into account in the simulation box, as the modal analysis is performed in the reference frame attached to the material. With the large deformations of the web, we estimate that around 10$\%$ of the relative variations of the eigenfrequencies are due to the geometric deformation ($\delta L /L \simeq 0.1$). In addition, silk is a highly nonlinear material, which suggests various possibilities for the tunability of the structure. The term $\delta T/T$ is also simplified here to linear prestress. This simplification may be justified with the following remark: at the beginning of the plastic zone, the apparent Young’s modulus of the silk is constant in the 0.15-0.25 strain range^27^, meaning that material nonlinear effects should be negligible in this zone. In conclusion, although the exact strain and stress states in the web cannot be derived, we will suppose that modal analysis can accurately describe its dynamical response, for a given prestress state.

### Modal imaging

To further study the vibration transmission properties of the web, an imaging method, based on a modal expansion and Green’s function composition^31^, allows us to place ourselves from the spider’s point of view. This approach is based on the fact that a spider has 8 legs and is extremely sensitive to vibrations^32–34^. By treating each leg as an independent sensor, the spider can accumulate the information from several modes of vibration of the web, excited following the impact of a prey.

The mode shapes $\phi _{in}$ (Fig.2) can be used *a posteriori* to compute virtual sources and estimate their *visibility* to the spider as a function of its position on the web. For a source located at point *i*, the broadband motion induced in a point indexed *j* can generally be expressed as^31,35–37^:

5 $$\begin{aligned} G_{ij} = \phi _{i n} \phi _{n j}^{\scriptscriptstyle \dag }, \end{aligned}$$

where $\phi _{i n}$ denotes the motion induced by the mode *n* at point *i*. This is a real function, representing a standing wave in space. As Eq. (5) remains true for complex mode shapes, we use here complex conjugate notation $^{\scriptscriptstyle \dag }$. We also remove time dependency in Eq. (5) and neglect damping effects, meaning that this equation is valid equivalently for particle displacement, velocity or acceleration. The number of modes n dictates the spatial resolution. With an infinite number of modes, the Green function tends to a Dirac function in space. With a finite number of modes, the spatial resolution decrease drastically. Although this approach does not attempt to model the temporal evolution of a real mechanical impact, focusing only on its kinematic consequences, the theoretical expansion in Eq. (5) remains exploitable in real experiments for active source localization^24^. The capability of the spider to detect sources can then be estimated using a composition of the Green’s function between the source *i*, a subset of points *r* and the set of points *j* of the domain defined by the web. This composition, according to the notations used in Eq. (5), is written as:

6 $$\begin{aligned} G_{ij} \longrightarrow G_{ir} G_{rj}= & {} \phi _{in}\phi _{n r}^{\scriptscriptstyle \dag }\phi _{rn'}\phi _{n'j}^{\scriptscriptstyle \dag } \nonumber \\= & {} \phi _{in} B_{nn'}\phi _{n' j}^{\scriptscriptstyle \dag } \end{aligned}$$

where $B_{nn'}$ is the so-called *visibility* matrix. If the implicit summation in Eq. (6) is performed on the whole domain, the spatial orthogonality of the eigenmodes implies that $B_{nn'}$ is an identity matrix. Instead, when only a limited number of points “*r*” is used for the composition of Eq. (6), an artificial coupling appears between the modes, affecting the reconstruction of the source image. In addition, the number of modes *n* dictates the spatial resolution. With an infinite number of modes, the Green function tends to a Dirac function in space. With a finite number of modes, the spatial resolution decreases drastically. In the case of the spider/spiderweb system, Eq. (6) is evaluated for a limited number of measurement points *r*, coinciding with the contact points of the eight legs and in our case using only sixteen modes. The expected spatial resolution is therefore imperfect, but sufficient for the purpose of prey localization. It is therefore possible to simulate the spider’s ability to localize prey as a function of its position on the web. To summarize, Eq. (6) combine two Green functions which correspond: i) to the vibration signal induced by the prey’s impact ($G_{ir}$) and ii) to the mechanical representation that the spider produces of the web from its leg locations ($G_{rj}$). The matrix $B_{nn'}$ may be seen as an autoMAC criterion^38^. It gathers the conditions for the source reconstruction from the spider’s point of view.

## Results

### Prey detection

**Figure 3:**
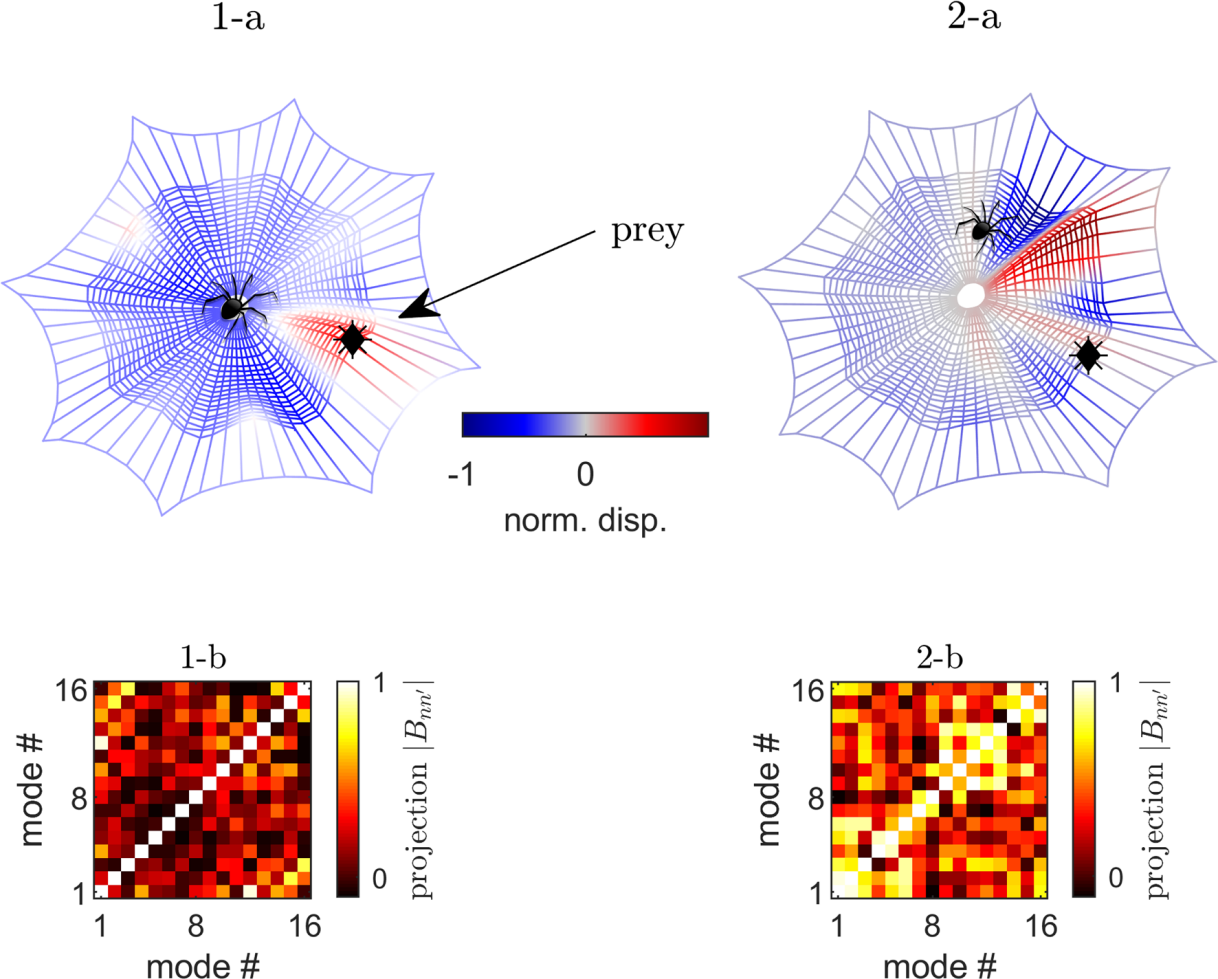
(Color online) Effect of the position of the spider on its detection capacity. Case 1, panel (**1-a**) the spider is in the center and the source (black dot) is correctly detected. The color code and the web deformation quantify the normalized displacement field, induced by the source, as reinterpreted by the spider. The absolute value of the visibility matrix $\vert B_{nn'} \vert$ in panel (**1-b**) shows small out-of-diagonal values. In Case 2, panel (**2-a**) the spider is off-center and the source is not correctly imaged. The visibility matrix (in absolute value) in panel (**2-b**) shows larger out-of-diagonal values, indicating cross-talk between modes. Pictures and maps are made with $\hbox {COMSOL}^{{\mathrm{TM}}}$ 6.0 and $\hbox {MATLAB}^{{\mathrm{TM}}}$ R2022b softwares. https://www.comsol.com/livelink-for-matlab.

Figure 3 shows the calculated imaging results (in terms of normalized displacements) for an off-center impact point in two cases, i.e. when the spider is located centrally in the web, and when its position is off-center. In both cases, the spider legs are chosen to lie around the spider location with a constant azimuthal distribution ($\simeq 2\pi /9$) and they are considered perfectly attached to the radial threads. The color map illustrates the reconstructed amplitude of the elastic source created by the impact of a prey with the vibration amplitude reported in the −1/1 range. As only 16 modes are used, the spatial resolution is better over the azimuthal direction than along the radial one, creating this red cone in Fig. 31-a. Increasing the number of modes could help to better localize the prey, but would also increase outer diagonal values in the $B_{nn'}$ matrix, leading to additional errors in the source reconstruction. In the first case, the prey impact point (indicated by a black dot) is faithfully reproduced, with a spatial uncertainty that is related to the finite number of modes used to build the image. Indeed, only one web aperture is highlight by the imaging process (in red on Fig. 31-a). In the second case, the prey position cannot be properly reconstructed with many apertures created by the imaging on Fig. 31-b. In Figs. 31-a and 2-a, the graphical representation of the spider is visual and is not included in the simulation. The positions of its eight legs are used as analysis points. A slight eccentricity of the web is accompanied by a deterioration of the virtual source reconstruction. The quality of the image can quantitatively be accessed with the $B_{nn'}$ matrix, defined in Eq. (6) and represented graphically in Fig. 31-b, 2-b for the two considered cases. Large non-diagonal values indicate cross-talk between modes and thus incorrect imaging of the source.

### Discussion

Although vibration sensing is by no means the only source of information for spiders in hunting prey, and possibly not even the most important, the results presented above suggest that spiders are capable of recognizing the different vibration modes supported by the web. The very structure of the web could be somehow related to the modal distribution required for optimal structural monitoring.

In particular, from the developed simulation box and proposed data analysis, we observe that the fundamental mode of the web does not contribute to the final image of the prey. From Fig. 2, one can see that if the fundamental mode is well isolated in frequency from other modes (around 40 Hz, in our case), the modal density subsequently increases, and can be estimated as around 1 mode / 2 Hz. This particular modal density could occur by design. Indeed, as the fundamental mode is not necessary to construct an image, it may be used only to capture the prey after impact, exploiting its large damping properties, without overlapping with higher modes that are used to locate the prey. This first mode may also be used for environmental monitoring. Indeed, it is the only mode that presents maximum vibration amplitude at the center and thus, at the spider location. Therefore, it may be used to amplify external sources of noise, increasing the spider sensitivity to its environment^9^.

The actual effect of the spider on the vibration response of the web is neglected in this work. The spider introduces additional mass to the system, but also additional stiffness, since its legs are much more rigid in flexion than the silk. Analysis of the modal participation factor associated for each mode indicates that the fundamental mode at 40Hz is the most affected by the mass of the spider, whereas the other modes may be more affected by the additional rigidity. Indeed, the web center corresponds to a maximum of vibration for the fundamental mode but is at a node location for all other modes. Thus the modeling of the spider is possible but would add complexity to the system and require the use of further hypotheses in its physical description, i.e., of the silk as pre-stressed truss elements and of the spider in terms of beams subjected to bending. This will be the object of a future study.

## Conclusion

In this work, we have constructed a qualitative mechanical model of a spider orb web, including prestress and geometrical nonlinearity. We have shown that this system acts as a multimodal transverse resonator system, naturally designed for prey impact detection. The resulting outputs of the modeling, such as resonance frequencies and prestress values within the structure are consistent with previously reported experimental data. Then, using modal expansion and Green’s function composition methods, we have proposed an imaging mechanism that could be used by the spider to exploit the vibration of its web, measured by its eight independent legs, to detect and localize prey. These results can be adapted both for practical applications in elastic wave imaging (e.g. real-time impact detection for structural health monitoring in frame-like structures) and material design problems. They also provide additional insight and understanding of the fascinating ways in which vibration phenomena are exploited in Nature.

## Data Availability

The datasets used and analyzed during the current study available from the corresponding author on reasonable request.
