# Author Correction: Prey localization in spider orb webs using modal vibration analysis

**DOI:** 10.1038/s41598-022-27004-1

**Published:** 2022-12-28

**Authors:** Martin Lott, Vinicius F. Dal Poggetto, Gabriele Greco, Nicola M. Pugno, Federico Bosia

**Affiliations:** 1 Department of Applied Science and Technology, Politecnico di Torino, Turin, Italy; 2 Laboratory for Bioinspired, Bionic, Nano, Meta Materials and Mechanics, University of Trento, Trento, Italy; 3 School of Engineering and Materials Science, Queen Mary University of London, Mile End Road, London, E1 4NS UK

Correction to: *Scientific Reports* 10.1038/s41598-022-22898-3, published online 09 November 2022

The original version of this Article omitted an affiliation for Nicola M. Pugno. The correct affiliations are listed below.

Laboratory for Bioinspired, Bionic, Nano, Meta Materials and Mechanics, University of Trento, Trento, Italy.

School of Engineering and Materials Science, Queen Mary University of London, Mile End Road, London E1 4NS, United Kingdom.

In addition, Martin Lott was incorrectly listed as a corresponding author. The correct corresponding author for this Article is Federico Bosia. Correspondence and request for materials should be addressed to federico.bosia@polito.it.

The original Article has been corrected.

